# *BIME2*, a novel gene required for interhomolog meiotic recombination in the protist model organism *Tetrahymena*

**DOI:** 10.1007/s10577-017-9563-y

**Published:** 2017-08-12

**Authors:** Anura Shodhan, Maria Novatchkova, Josef Loidl

**Affiliations:** 1 Department of Chromosome Biology, Vienna Biocenter, University of Vienna, 1030 Vienna, Austria; 2 Laboratory of Biochemistry and Molecular Biology, National Cancer Institute, Bethesda, MD 20892 USA; 3 Institute of Molecular Biotechnology of the Austrian Academy of Sciences and Research Institute of Molecular Pathology, 1030 Vienna, Austria

**Keywords:** Meiosis, Chromosome pairing, Crossover, Recombination, Double-strand break

## Abstract

Meiotic recombination is initiated by DNA double-strand breaks (DSBs). Most DSBs are converted into nonreciprocal exchanges (gene conversions) or crossovers (COs) between sister chromatids. Only a minority of DSBs are processed toward interhomolog COs, the precursors of the chiasmata that connect homologous chromosomes. Dmc1, the meiosis-specific paralog of the universal recombination protein Rad51, is required for interhomolog COs; in its absence, univalents are primarily formed. Here, we report a ciliate-specific novel meiotic gene, *BIME2*, which also promotes interhomolog crossing over. In the *bime2*Δ mutant, DSBs are formed and repaired normally, but bivalent formation is strongly reduced. Bime2 protein forms foci on chromatin during meiotic prophase, and chromatin localization of Bime2 and Dmc1 is largely interdependent. Bime2 distantly resembles budding yeast Rdh54/Tid1 and the vertebrate Rad54B helicases and may have similar functions in promoting or stabilizing Dmc1 nucleoprotein filaments.

**Electronic supplementary material:**

The online version of this article (doi:10.1007/s10577-017-9563-y) contains supplementary material, which is available to authorized users.

## Introduction

Meiotic recombination enables the formation of interhomolog crossovers (COs). In this process, numerous DNA double-strand breaks (DSBs) are generated to ensure proper homology searching and homologous pairing. However, only a small subset of DSBs are converted into COs and chiasmata, necessary for the orderly segregation of homologous chromosomes and genetic recombination. Most DSBs are repaired by nonreciprocal exchange (gene conversion) or recombination between sister chromatids (see Goldfarb and Lichten 2010; Chapman et al. 2012). To ensure that sufficient COs are formed between homologs, mechanisms act to suppress the more readily occurring Rad51-dependent intersister recombination events (Niu et al. 2009) or actively promote interhomolog recombination. One interhomolog-promoting factor is Dmc1, a meiosis-specific paralog of the ubiquitous Rad51 recombinase (see Brown and Bishop 2015), which performs better in exchanging homologous DNA molecules with similar but not identical DNA tracts (see Howard-Till et al. 2011; Lee et al. 2015). The strand exchange activities of Rad51 and Dmc1 are supported by numerous proteins that facilitate and stabilize their association with single-stranded DNA (ssDNA) and promote homologous strand invasion and heteroduplex formation (see Brown and Bishop 2015). One such factor is Tid1/Rdh54, which interacts with both proteins, but is believed to specifically cooperate with Dmc1 in meiosis (Nimonkar et al. 2012).

*Tetrahymena thermophila* is a versatile protist model organism with a history in groundbreaking discoveries, such as self-splicing introns, histone-modifying enzymes, and telomeres and telomerase (see Ruehle et al. 2016). Also, apart from fungal, animal, and plant model systems, *Tetrahymena* is the organism with the best-studied meiosis (see Loidl 2016). *Tetrahymena* meiosis is remarkable for its simplicity, the absence of a synaptonemal complex (SC), and the extreme stretching of meiotic prophase nuclei in response to Spo11-induced DSBs (Chi et al. 2014; Mochizuki et al. 2008; Loidl and Lorenz 2016). Most eukaryotes use two major pathways to form COs: The class I pathway involves SC formation and ZMM (**Z**ip1/2/3/4, **M**sh4/5, and **M**er3) proteins and generates interfering (i.e., mutually suppressing) COs. The class II pathway is largely ZMM-independent and produces non-interfering COs (de los Santos et al. 2003). In contrast, *Tetrahymena* uses a single-mixed pathway, involving the ZMM proteins Msh4, Msh5, and a protein similar to Zip3 (Shodhan et al. 2014; Shodhan et al. 2017). As in most other organisms, only a fraction of DSBs are converted into homolog-directed COs. However, unlike budding yeast and probably other SC-possessing organisms, where the chromosome axis-associated kinase Mek1 and the axial element components Red1 and Hop1 are involved in inhibiting Rad51-dependent intersister recombination (e.g., Thompson and Stahl 1999; Schwacha and Kleckner 1997; Niu et al. 2009; Chuang et al. 2012; Hollingsworth et al. 1995), *Tetrahymena* depends solely on the interhomolog preference of Dmc1 (Howard-Till et al. 2011). Here, we report a novel protein which, together with Dmc1, ensures interhomolog recombination in *Tetrahymena*.

## Materials and methods

### Strains and cell culture

Cells were cultured at 30 °C using standard methodology (Orias et al. 2000) and were made competent for mating by starvation in 10 mM Tris-HCl (pH 7.4) for at least 16 h. Meiosis was induced by mixing starved cultures of B2086 (mating type II) and Cu428 (mating type VII) wild-type or derivative mutant strains at equal densities (∼ 2 × 10^5^ cells/ml).

### Somatic gene knockout and protein tagging

For somatic gene knockout, (almost) all of the ~ 50 copies of a target gene in the polyploid somatic macronucleus must be replaced with a deletion cassette carrying an antibiotic resistance marker. Moreover, to investigate the effects of gene inactivation in meiosis, the gene must be deleted in both mating partners because mating cells can share gene products (McDonald 1966). For *BIME2* deletion, 1767 bp of the open reading frame was replaced with a construct carrying a neomycin resistance marker (Supplemental information S1a), by homologous recombination of flanking regions (Cassidy-Hanley et al. 1997; Mochizuki 2008). Knockout lines were selected by culture in medium with increasing concentrations of the neomycin analog paromomycin. Complete gene replacement was confirmed by Southern hybridization to a restriction fragment spanning the gene locus (Supplemental information S1b).

A Bime2-HA-tagged strain was created by fusing the HA sequence to the 3′ end of the *BIME2* open reading frame (Supplemental information S1c). Construction of *dmc1*RNAi (Howard-Till et al. 2011) and *spo11*RNAi strains (Lukaszewicz et al. 2013) was done as previously reported. Mating of the Bime2-HA-tagged strain to *dmc1*RNAi and *spo11*RNAi cells led to depletion of the respective endogenous protein and HA-tagged Bime2 expression in both partners as a result of the cytoplasmatic exchange of RNA molecules and proteins between mating cells (McDonald 1966). A strain expressing mCherry-tagged histone H3 was kindly provided by Dr. Kensuke Kataoka (Natl Inst. Basic Biol., Okazaki, JP).

### Cytological preparation, staining and microscopy

For 4′,6-diamidino-2-phenylindole (DAPI) staining, cells were fixed in formaldehyde and spread on a slide (Mochizuki et al. 2008). For Bime2 and Dmc1 localization studies, cells were pretreated with Triton X-100 to remove protein not bound to chromatin (Howard-Till et al. 2011), and then, primary and fluorescent secondary antibodies were applied. Dmc1 was detected using a commercial antibody (51RAD01 mouse monoclonal, NeoMarkers, Fremont, CA). Samples on slides were mounted in anti-fading solution containing DAPI as a stain for chromatin and were evaluated by fluorescence microscopy using appropriate filters. Image stacks were recorded using MetaVue software (Molecular Devices, Sunnyvale, CA) and deconvolved. A Schaudinn fixation plus Giemsa staining method (Bruns and Brussard 1981; Shodhan et al. 2014) was used to release nuclei from cells, and the resulting flattened, well-separated chromosomes were inspected under bright-field microscopy.

### DSB detection

To analyze DSB-dependent DNA fragmentation, DNA was isolated from cells at different time points after induction of meiosis. DNA fragments were separated by pulsed-field gel electrophoresis and visualized by Southern hybridization to a radiolabeled probe specific to the germline nucleus (for details, see Lukaszewicz et al. 2010).

### Protein co-immunoprecipitation

For co-immunoprecipitation (co-IP) experiments, cells were harvested at ~ 3.5-h post-meiotic induction (at the stage with maximum nuclear elongation), washed, resuspended in ice-cold Tris lysis buffer (100 mM Tris-Base, Tris-HCl, 1 M KCl, 1 M MgCl, 1% Triton X-100, 0.01 M PMSF, pH 7.5), and ground in a Dounce homogenizer. The cell lysate was clarified, filtered, and incubated with anti-HA magnetic beads (Thermo Fisher Scientific, Waltham, MA) for 2 h at 4 °C. (For details of the procedure, see Shodhan et al. 2017.) After washing, two thirds of the protein-loaded beads were analyzed by mass spectrometry and protein eluted from the remaining third was analyzed by Western blotting.

## Results and discussion

### Bime2 is important for bivalent formation

We identified *BIME2* (BIvalents in MEiosis 2; TTHERM_00530659—see http://ciliate.org/) in a reverse genetic screen, in which genes exclusively expressed during sexual reproduction (conjugation) were knocked out. *BIME2* expression is highest at around 2–4 h after induction of meiosis (Xiong et al. 2012); http://tfgd.ihb.ac.cn/), i.e., when homologous pairing and recombination occur (see Loidl and Lorenz 2016). *BIME2* expression is controlled by the conjugation-specific cyclin Cyc2, and *CYC2* deletion led to the strongest repression of *BIME2* transcription compared with all other meiotic genes (Xu et al. 2016).

**Fig. 1:**
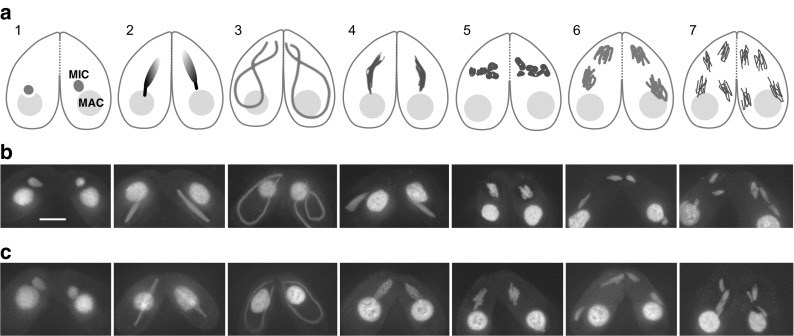
Stages of *Tetrahymena* meiosis. **a** Two cells of different mating types mate upon starvation. Each cell has a polyploid somatic macronucleus (MAC) and a diploid germline micronucleus (MIC). Only the germline nucleus undergoes meiosis. The somatic nucleus is degraded after meiosis, whereas the products of micronuclear meiosis undergo reciprocal fertilization, and progeny germline and somatic nuclei are formed from the zygotic nuclei. 1. Initiation of synchronous closed meioses in the two mating partners. 2. Early prophase. DSBs are formed, meiotic nuclei begin to elongate. 3. Mid-prophase. Nuclei elongate to about twice the cell length. Chromosomes are arranged in parallel bundles within the elongated nuclei, with centromeres assembled near one tip and telomeres at the opposite tip. Homologous pairing takes place. 4. Late prophase. Nuclei shorten, DSBs are repaired and COs are formed. 5. Five condensed bivalents appear. 6. First meiotic division. 7. Second meiotic division. (For detailed descriptions of cytological stages and the corresponding events of molecular recombination, see e.g. Loidl and Lorenz 2016; Shodhan et al. 2014; Loidl et al. 2012.) **b** DAPI-stained wild-type meiosis. **c** DAPI-stained *bime2*Δ meiosis. Scale bar: 10 μm

Cytological analysis showed that *bime2*Δ cells undergo all stages of meiosis (Fig. 1). However, after anaphase II, all four haploid nuclei are degraded in about half (52%, *n* = 100) of *bime2*Δ meiotic cells, and none of the mating pairs produced viable sexual progeny (compared with 72% of wild-type mating pairs; *n* = 150 each).

**Fig. 2:**
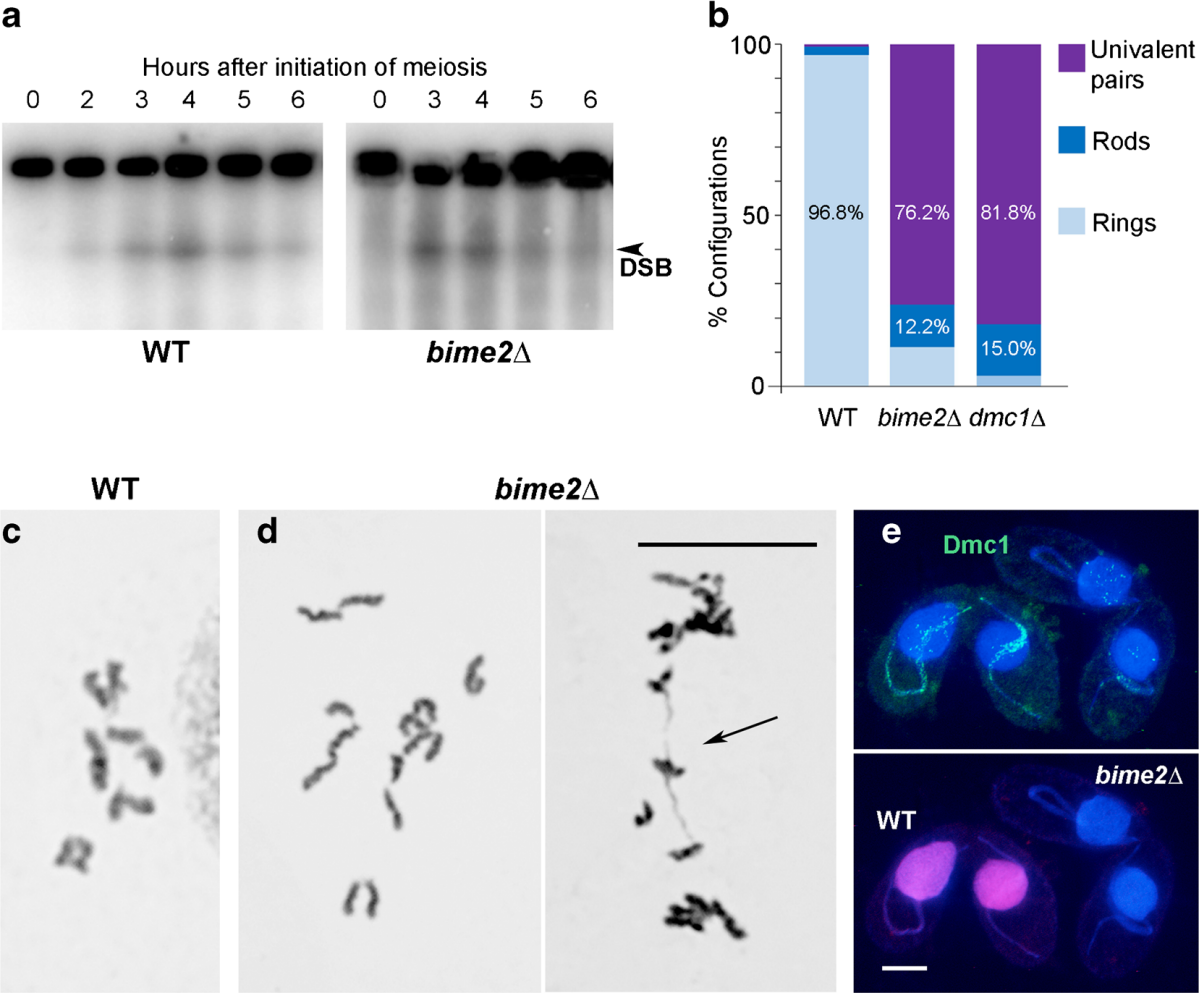
Deletion of *BIME2* prevents chromosome pairing and inhibits Dmc1 chromatin localization. **a** Southern hybridization of DSB-dependent chromosome fragments separated by pulsed-field gel electrophoresis using a probe specific to the germline nucleus. DSB formation is similar in wild-type and *bime2*Δ cells. **b** Ring bivalents are mainly formed in the wild type, whereas univalents and rare bivalents are formed in *bime2*Δ and *dmc1*Δ meiosis. In wild type and *bime2*Δ, 500 configurations (bivalents or pairs of univalents) were counted; in *dmc1*Δ, 400 configurations were counted. **c**, **d** Examples of Giemsa-stained diakinesis-metaphase I wild-type (**c**) and *bime2*Δ (**d**) nuclei (arrow indicates a rod bivalent). **e** Chromatin-associated Dmc1 foci are present in elongated prophase nuclei in wild-type mating cells (distinguished by mCherry-tagged histone—magenta), but foci numbers are greatly reduced in *bime2*Δ mating cells. (Foci in somatic nuclei represent Rad51, which is also recognized by the anti-Dmc1 antibody). Scale bars: 10 μm

In *Tetrahymena*, DSBs trigger the extreme elongation of meiotic prophase nuclei (Mochizuki et al. 2008). In *bime2*Δ cells, nuclear elongation is normal, suggesting that DSBs are formed during meiosis. DSB formation in *bime2*Δ cells was confirmed by the detection of transient germline chromosome fragments by pulsed-field gel electrophoresis, similar to wild-type cells (Fig. 2a).

To investigate the cause of infertility, we analyzed chromosome pairing by Schaudinn fixation followed by Giemsa staining, which releases diakinesis-metaphase I chromosomes from cells. We found that bivalent formation was strongly reduced in *bime2*Δ compared to wild type. In the wild type, 0.6% of chromosome pairs formed univalents, 2.6% formed rod bivalents (in which one chromosome arm is connected), and 96.8% formed ring bivalents (in which both arms are connected). However, in *bime2*Δ meiosis, we found that 76.2% of chromosome pairs formed univalents, 12.2% formed rod bivalents, and only 11.6% formed ring bivalents (Fig. 2b, c). If we assume that ring bivalents have at least two COs (one in each arm) and rod bivalents have one, the number of COs in *bime2*Δ is estimated at 18% of the wild-type number. This strong reduction in COs is reminiscent of *dmc1*Δ meiosis (Fig. 2b and Howard-Till et al. 2011).

After DSB resection, the association of Dmc1 with single-stranded 3′ overhangs at DSB ends enables homology searching. More than 150 Dmc1 foci are visible in elongated nuclei in wild-type strains (Howard-Till et al. 2011; Lukaszewicz et al. 2015). In contrast, in the absence of Bime2, 80% (*n* = 250) of elongated nuclei completely lacked a Dmc1 signal, while the rest showed a faint or diffuse Dmc1 signal (Fig. 2e). To enable a direct side-by-side comparison, *bime2*Δ and wild-type mating cells were mixed just before fixation and then stained together on the same slide. To allow their discrimination, cells expressing mCherry-tagged histone were used as wild type (Fig. 2e). Together, the reduced chromatin localization of Dmc1 in the absence of Bime2 and the similar degree of reduction in bivalent formation in *bime2* and *dmc1* mutants suggest that Bime2 and Dmc1 cooperate in homologous CO formation.

### Bime2 localizes to meiotic nuclei in a DSB-dependent manner

**Fig. 3:**
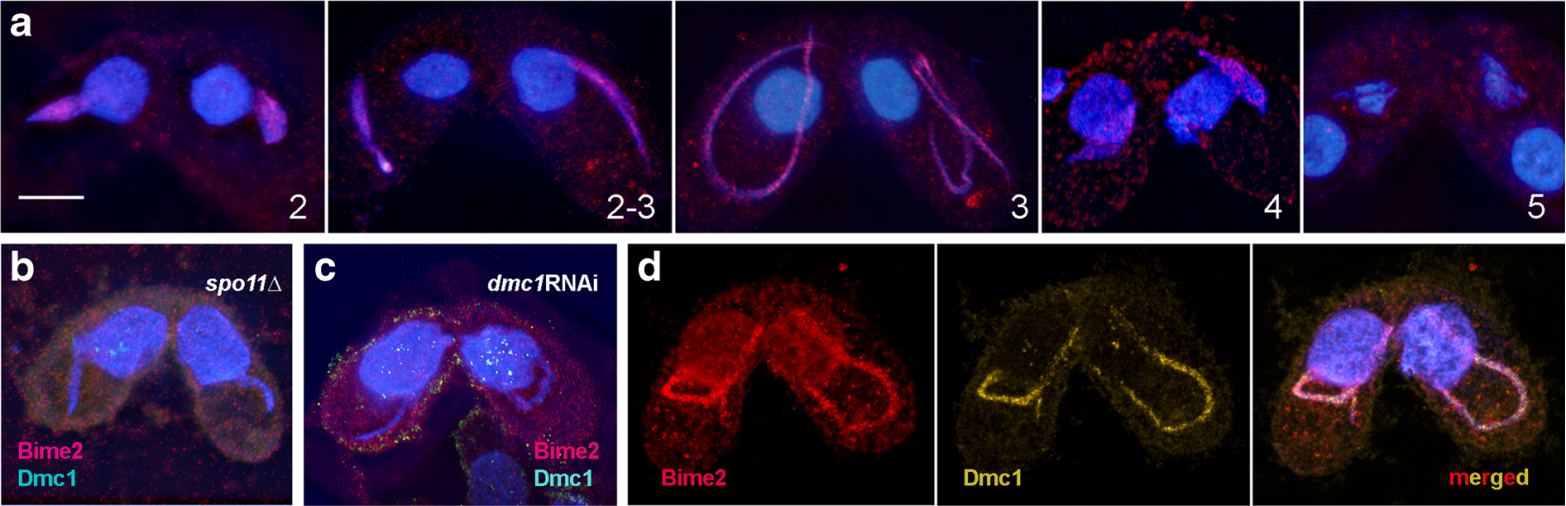
Bime2 localization. **a** HA-tagged Bime2 (red) localizes to the chromatin of meiotic nuclei from the start of nuclear elongation to the end of prophase. It is undetectable in metaphase I. (Numbers refer to the stages in Fig. 1a.) **b** Similar to Dmc1, Bime2 localization is Spo11 dependent. **c** Bime2 foci are not formed in Dmc1-depleted cells. Preparations were co-stainined for Dmc1—the absence of Dmc1 in meiotic germline nuclei confirms the efficiency of RNAi-mediated Dmc1 knockdown. Foci seen in somatic nuclei represent antibody cross-reactivity with Rad51. For Dmc1 localization in wild-type cells, see Fig. 2e. **d** Co-staining of Bime2-HA and Dmc1. Although both proteins form foci on the chromatin of meiotic prophase nuclei, they do not colocalize. Chromatin was stained with DAPI (blue). The bloated appearance of nuclei is caused by detergent treatment to remove non-chromatin-bound proteins. Scale bar: 10 μm

The subcellular localization of HA-tagged Bime2 was determined. Mating the Bime2-HA strain to a *bime2*Δ strain rescued the mutant phenotype (~ 85% ring bivalents were observed instead of ~ 10% in *bime2*Δ; *n* = 200 cells). Thus, the tagged protein was considered functional. Bime2 could only be seen during meiotic prophase: Bime2-HA foci first became visible in slightly elongated nuclei and disappeared by metaphase I (Fig. 3a). As these foci were seen in detergent-treated preparations in which non-chromatin-bound proteins are removed (Howard-Till et al. 2011), we conclude that they represent chromatin-associated Bime2. Bime2 localization was dependent on DSBs, since foci were absent in Bime2-HA × *spo11*RNAi mating cells (Fig. 3b). Next, *dmc1*RNAi cells were mated with Bime2-HA cells to test whether Bime2 localization is Dmc1 dependent. We found that Bime2 was always absent in mating pairs lacking Dmc1 (Fig. 3c). Because Bime2 localization is dependent on Dmc1 and Dmc1 localization is partially dependent on Bime2 (see above), it might be reasonable to assume that these two proteins colocalize in meiotic nuclei. However, double immunostaining of Bime2-HA and Dmc1 failed to show a complete overlap of Bime2 and Dmc1 foci (Figs. 3d and S2). It is possible that the two proteins occupy adjacent positions, but the large number of foci precluded a detailed analysis of spatial relationships.

To test for a possible interaction between Bime2 and Dmc1, Bime2-HA immunoprecipitation (IP) was performed and co-precipitating proteins were analyzed by Western blotting. However, Dmc1 was not detected (data not shown). Mass spectrometry showed that Bime2 was enriched in the Bime2-HA pulldown (log2 LFQ ratio Bime2-HA/Bime2-untagged = 12), but neither Dmc1 nor any other protein known to be involved in DSB repair or meiotic recombination was a significant hit (data not shown). Similarly, mass spectrometry analysis of a reciprocal Dmc1 IP did not identify Bime2 as a co-precipitating protein (Miao Tian, unpublished). We, therefore, conclude that chromatin localization of Dmc1 and Bime2 is mutually promoted but does not involve strong direct interaction.

### Bime2 is distantly related to Rad54B and Rdh54/Tid1 proteins

**Fig. 4:**
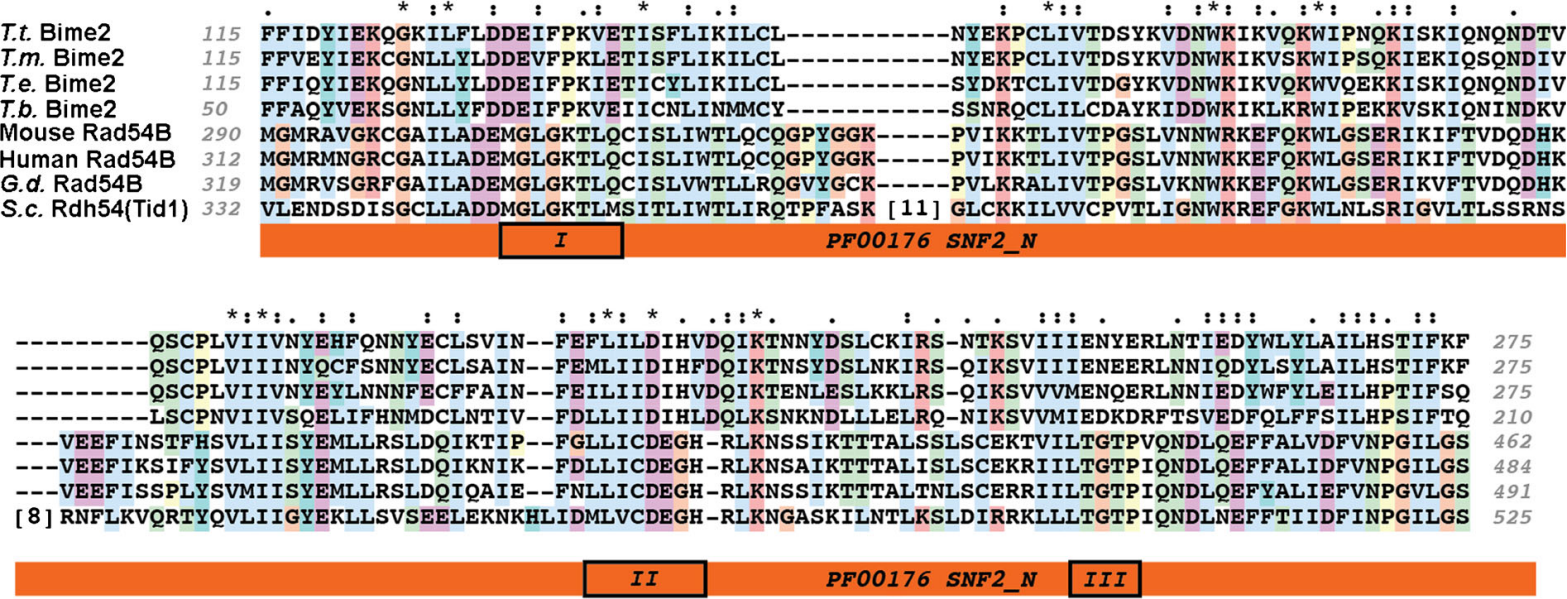
Multiple partial sequence alignment showing the region of the highest sequence similarity between *Tetrahymena thermophila* (*T.t.*) Bime2 proteins and representatives of the Rad54B family: mouse, human and chicken (*Gallus domesticus* (*G.d.*)) Rad54B and budding yeast (*Saccharomyces cerevisiae* (*S.c.*)) Rdh54/Tid1. The aligned Bime2 sequence segment was selected to include the region with significant similarity to the PANTHER family Rad54B/PTHR10799:SF918 (HMMscore versus PANTHER v11.1—*E* = 3.7e-09) and Pfam family PF00176/SNF2_N. Characteristic helicase sequence motifs, Motifs I (Walker A), II (DExx), and III, reported to form the primary ATP binding site in the active site cleft (Dürr et al. 2005) are marked, but appear not to be functionally conserved in Bime2. Bime2 has clear homologs only in other *Tetrahymena* species (*T. malaccensis* (*T.m.*), *T. elliotti* (*T.e.*), and *T. borealis* (*T.b.*)). The alignment was generated using MUSCLE v3.8.31 (Edgar 2004) and visualized using Clustalx v2.1 (Thompson et al. 1997)

*Tetrahymena* Bime2 proteins were predicted to contain a PF00176.22/SNF2_N domain (*E* = 2.2e-04, HHpred using Bime2 orthologs from four *Tetrahymena* species as input). Further sequence analysis revealed significant similarity between Bime2 and the PANTHER Rad54B/PTHR10799:SF918 subfamily in the Rad54-like subgroup of SNF2 helicase-related proteins (*E* = 3.7e-09. PANTHER db v11.1 hmm Score) (Mi et al. 2017) (Fig. 4). Bime2 was also a significant hit (*E* = 0.001) in a reciprocal search of the *Tetrahymena* proteome using the Rad54B/PTHR10799:SF918 profile. However, in the same search, more than 20 other *Tetrahymena* proteins had greater similarity to the profile; the top hit TTHERM_00237490p is annotated as Rad54 in the Tetrahymena genome database (http://ciliate.org/). The Bime2 ATPase domain shows divergence from canonical helicase motifs that are typically conserved in the SNF2 helicase-like family, suggesting that it might lack ATPase activity, and resulting in the weaker support in the reciprocal search. However, none of the closer family members in *Tetrahymena* are expected to have a meiosis-specific function in interhomolog recombination because all are ubiquitously expressed (http://tfgd.ihb.ac.cn/).

Rad54 has important functions in mitotic and meiotic DSB repair (Nimonkar et al. 2012; Arbel et al. 1999; Shinohara et al. 2000; Schmuckli-Maurer and Heyer 2000): It is involved in ATP-dependent chromatin remodeling during homology searching and D-loop formation (Petukhova et al. 1998; Jaskelioff et al. 2003; Solinger et al. 2001), heteroduplex extension (Bugreev et al. 2006), and Rad51 turnover or removal from dsDNA (Solinger et al. 2002; Agarwal et al. 2011). However, Rad54 also has ATP-independent functions, such as the stabilization of Rad51 nucleofilaments on ssDNA during DNA repair (Mazin et al. 2003; Agarwal et al. 2011). The budding yeast and mammalian Rad54 paralogs, Rdh54/Tid1, and Rad54B, respectively, support both Rad51 and Dmc1 nucleofilament formation in mitosis and meiosis, but in meiosis, they preferentially promote Dmc1-mediated interhomolog recombination (Brown and Bishop 2015; Sarai et al. 2006; Shinohara et al. 1997). Bime2 shows considerable sequence divergence from other Rad54 family members with a functional ATPase domain, which makes it difficult to prove orthology of Bime2 to Rad54 or Rdh54/Tid1. Nevertheless, its meiosis-specific expression and pro-CO activity, along with the mutual promotion of chromatin localization by Bime2 and Dmc1, suggest that these two proteins cooperate to promote interhomolog vs. intersister COs.

## Electronic supplementary material

ESM 1 (PDF 146 kb)..

ESM 2 (PDF 174 kb)..

## Abbreviations

DSB: Double-strand break
CO: Crossover
SC: Synaptonemal complex
FISH: Fluorescence in situ hybridization
co-IP: Co-immunoprecipitation
