# Supplementary material for: BIME2, a novel gene required for interhomolog meiotic recombination in the protist model organism Tetrahymena
Source: Chromosome Res. 2017 Aug 12;25(3):291–8. doi: 10.1007/s10577-017-9563-y (PMC5662671; doi:10.1007/s10577-017-9563-y)
Supplement: Supplementary file 1 — (PDF 146 kb). [file 10577_2017_9563_MOESM1_ESM.pdf]

Supplemental Information 1

a Schematic of the knockout procedure

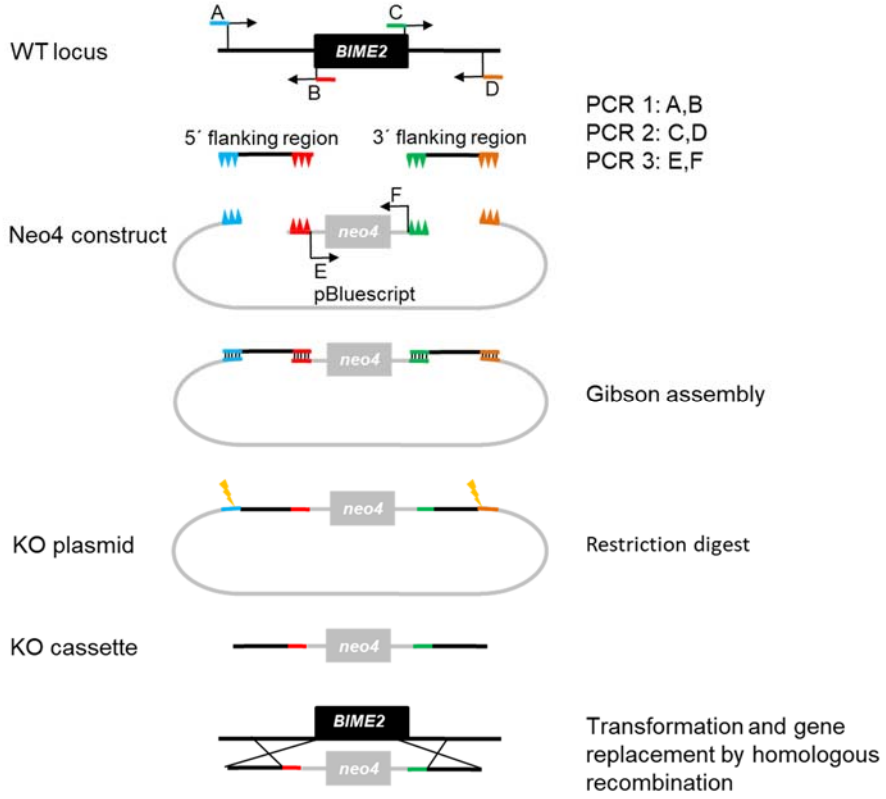

Primers for *bime2* knockout construct:

A: ctctagaactagtggatcccccgggccttttatattagcaaaccagc

B: cctgcagcccgcctttggttgaattgcc

C: aatgcagcccccttagactctactttgattgattattttatatc

D: ctctagaactagtggatcccccgggcttatgctccttacatttg

E: aaccaaaggcgggctgcaggaattcgatag

F: gagtctaagggggctgcatttttccagtaaaaatttg

b Southern hybridization for testing the *bime2* knockout (KO):

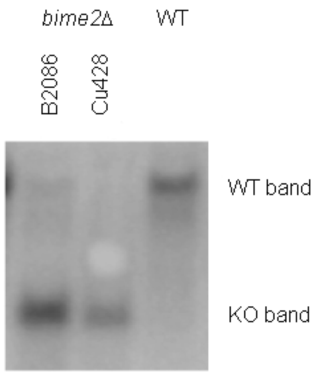

Southern hybridization showed that the *bime2* deletion was complete in both mating types. Genomic DNA was digested with EcoRI and hybridized with a probe that recognizes the 3’ UTR of *BIME2*.

Replacement with the knockout (KO) cassette produces a ~2.6 kb fragment, wheras the length of the WT fragment is ~6.1 kb.

c Primers for *BIME2*-HA tagging construct:
BIME2-HA5´FW_overlapping vector: ctctagaactagtggatccccaaacaaacaaaagtagagaaaatc
BIME2-HA5’FW_overlapping HANeo4: gataggatccagatataaaataatcaatcaaagtagagtc
HAFW_overlapping BIME2: attttatatctggatcctatccttatgatgttc
Neo4RV_overlapping BIME2: tactgactacaagcttgatatcgaattcagatc
BIME2-HA3’FW_overlapping HANeo4: tatcaagcttgtagtcagtaagatattcagtc
BIME2-HA3’RV_overlapping vector: ctctagaactagtggatcccgtgttttacttgttggttacg
The tagging construct was produced by Gibson assembly in the same way as the KO construct.
