# Supplementary material for: BIME2, a novel gene required for interhomolog meiotic recombination in the protist model organism Tetrahymena
Source: Chromosome Res. 2017 Aug 12;25(3):291–8. doi: 10.1007/s10577-017-9563-y (PMC5662671; doi:10.1007/s10577-017-9563-y)
Supplement: Supplementary file 2 — (PDF 174 kb). [file 10577_2017_9563_MOESM2_ESM.pdf]

Supplementary information S2

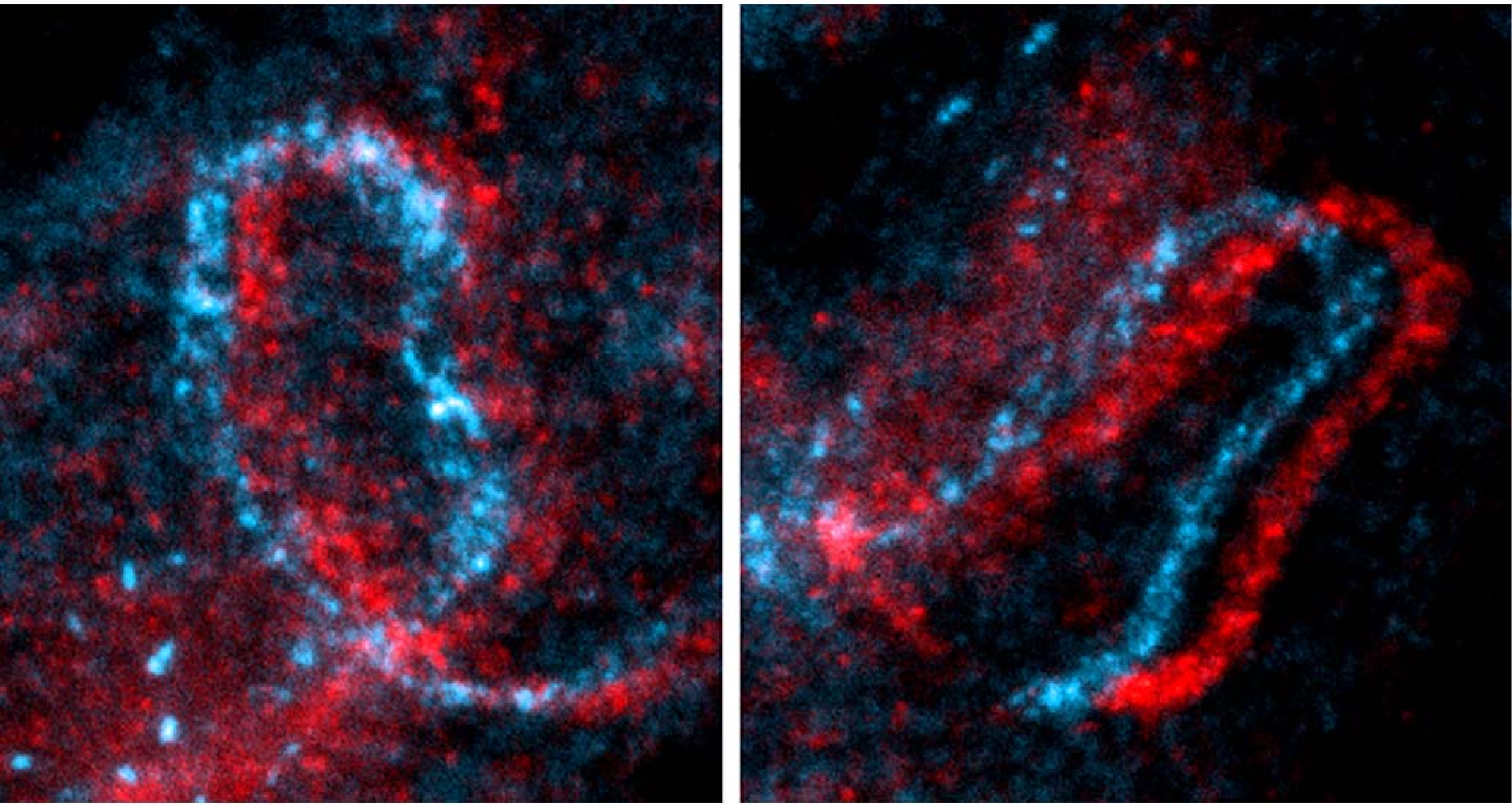

Examples of Bime2 (HA-tagged, red) and Dmc1 (immunostained with an anti-Rad51/Dmc1 antibody, cyan) foci in wild-type meiotic nuclei obtained from laser scanning microscopy.
The two images are offset along the X-axis. Foci produced by the two proteins do not perceptibly overlap.
